# Supplementary figures and images for: Parents’ smoking onset before conception as related to body mass index and fat mass in adult offspring: Findings from the RHINESSA generation study
Source: PLoS One. 2020 Jul 6;15(7):e0235632. doi: 10.1371/journal.pone.0235632 (PMC7337347; doi:10.1371/journal.pone.0235632)

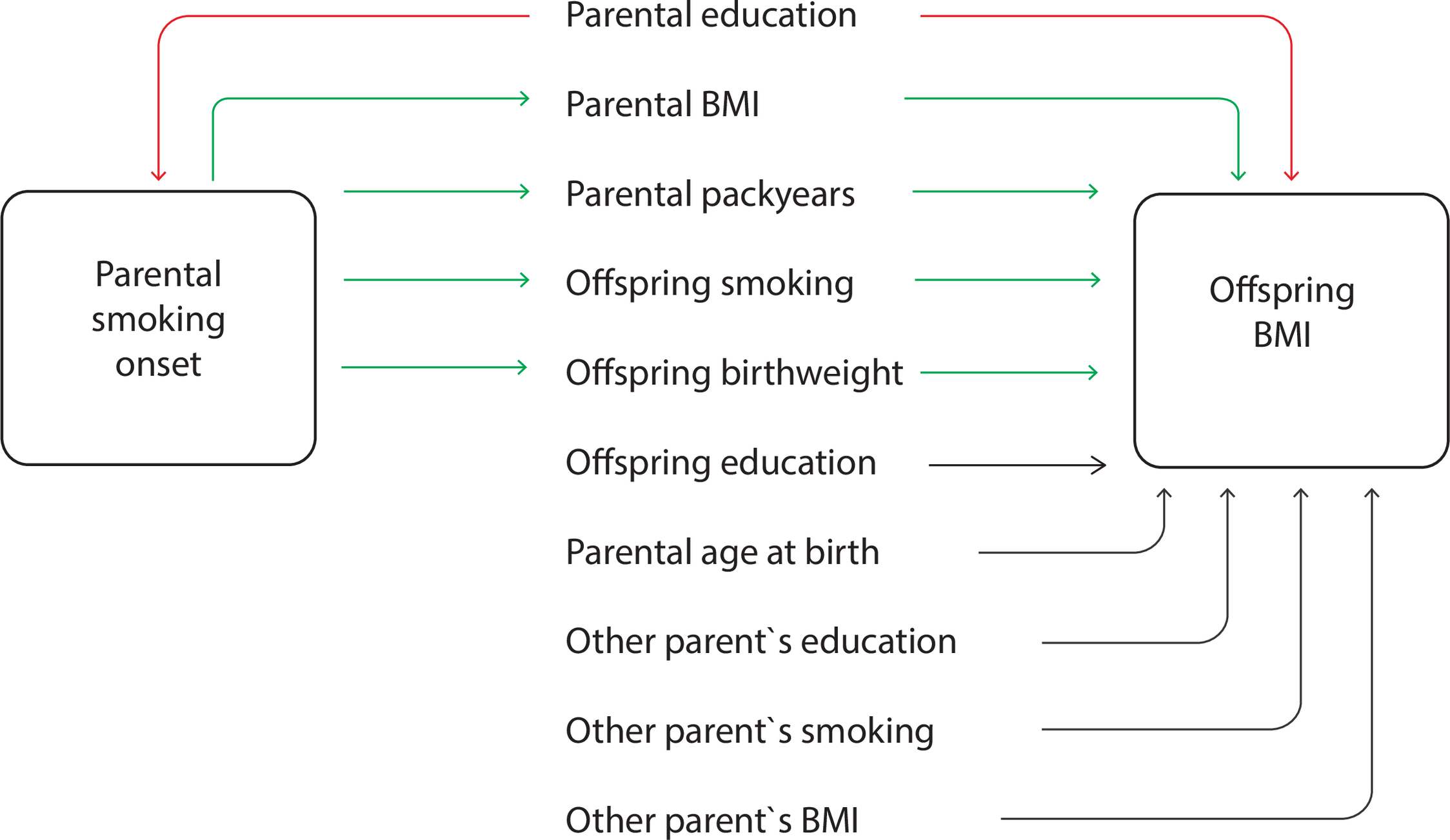

Supplement: S1 Fig — The figure presents covariates considered to be included in the statistical model. (TIF) [file pone.0235632.s001.tif]

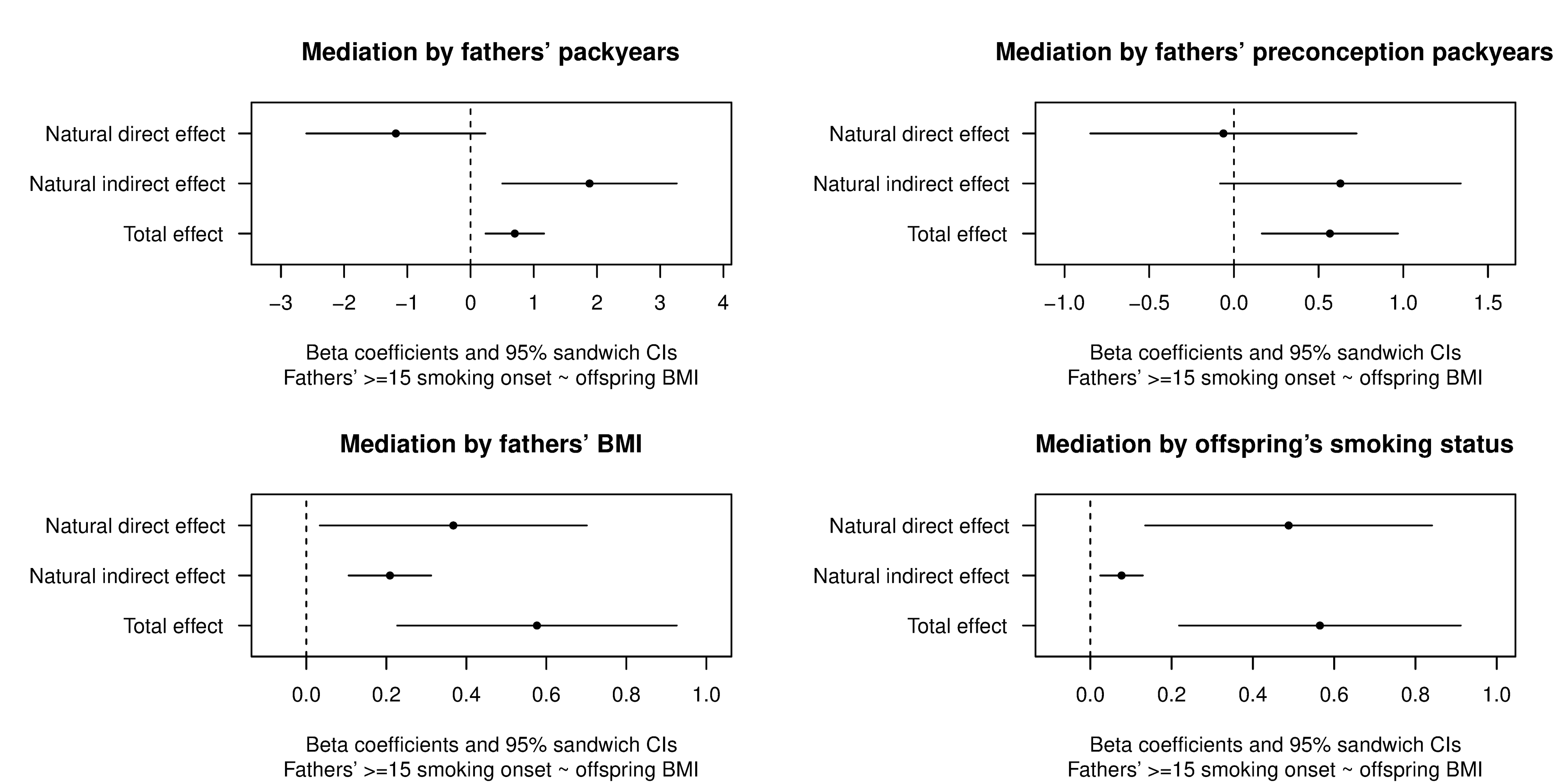

Supplement: S2 Fig — Analyses reveal full mediation by fathers’ pack years and partial mediation by fathers’ BMI and offspring’s own smoking status. There is no mediation via fathers’ preconception accumulative smoking. (TIF) [file pone.0235632.s002.tif]

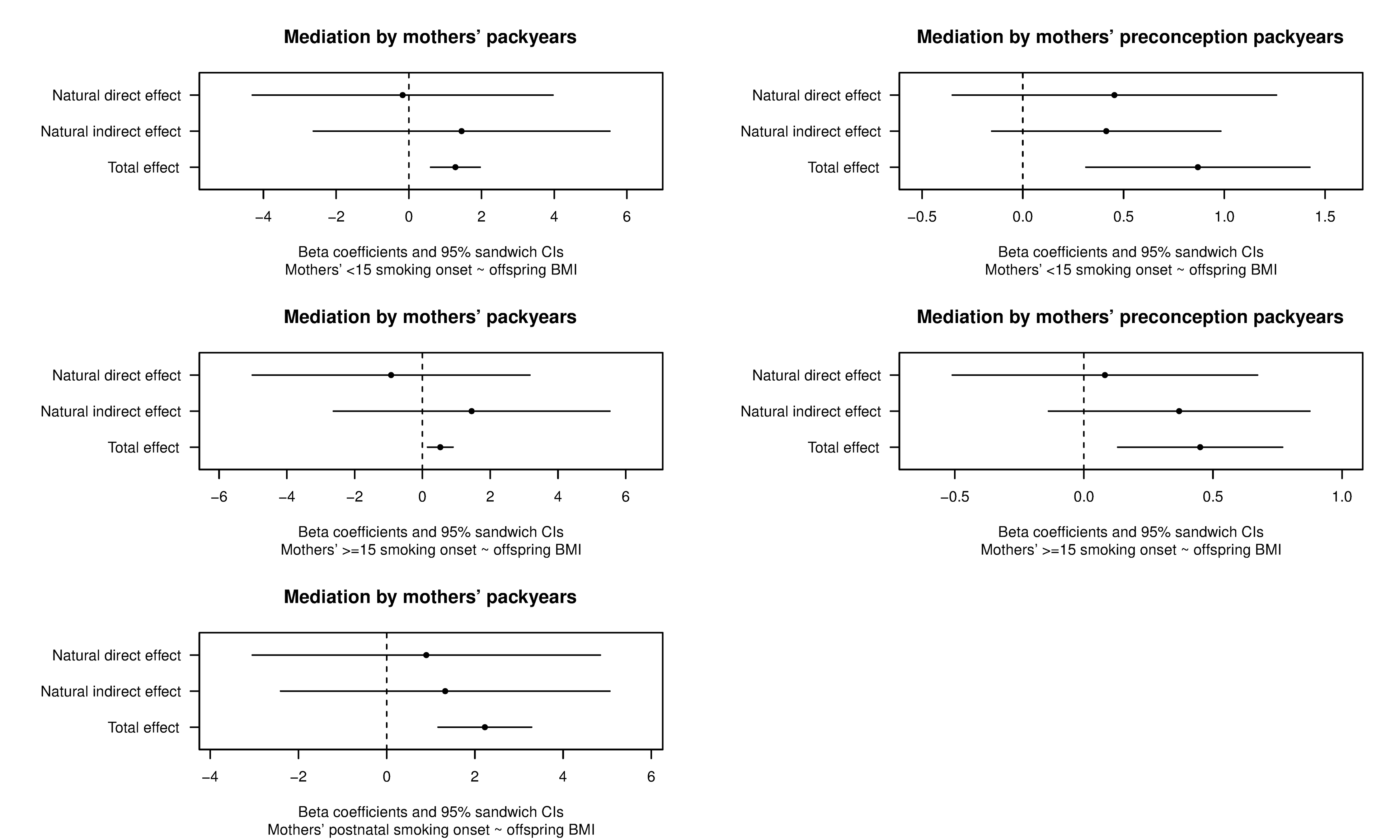

Supplement: S3 Fig — (TIF) [file pone.0235632.s003.tif]

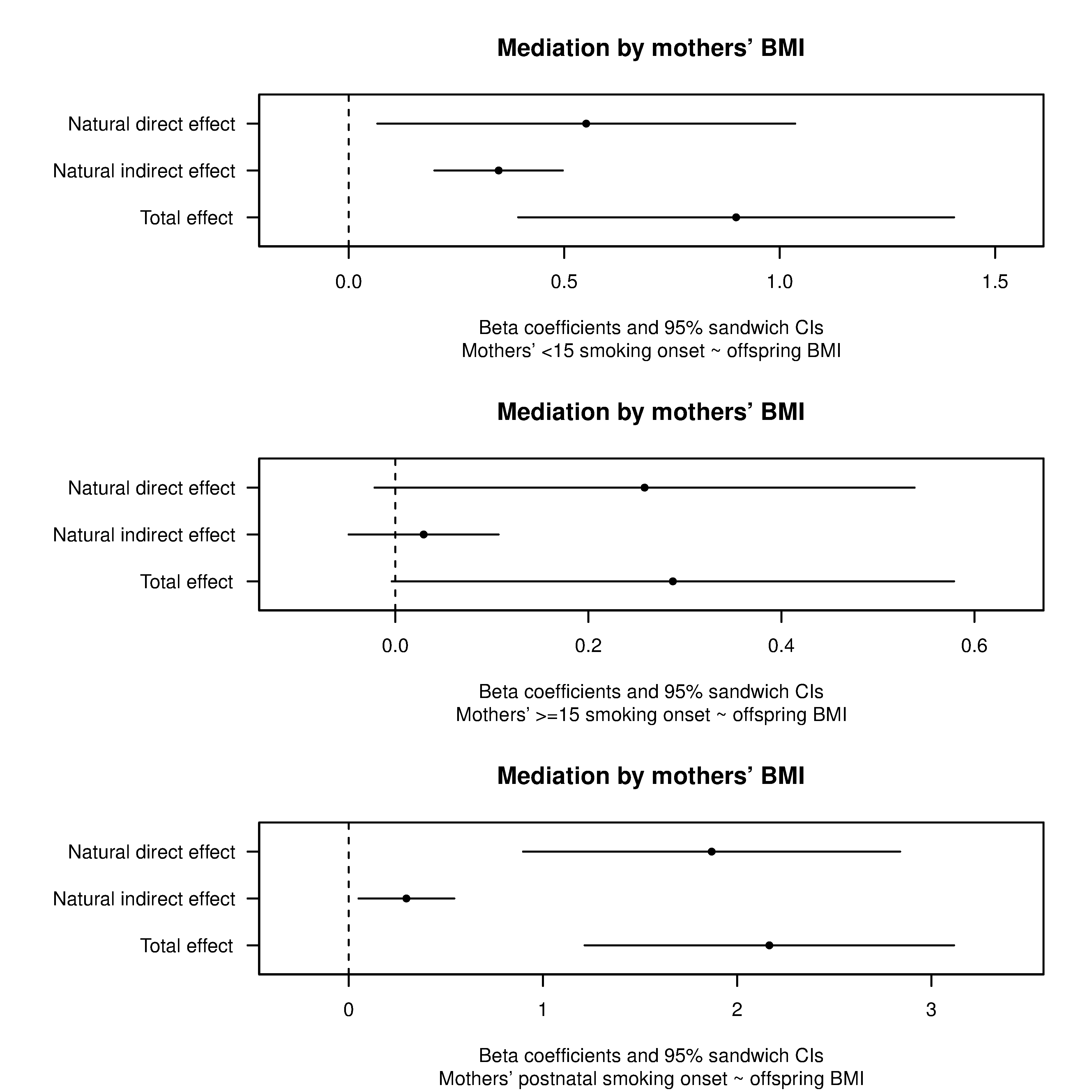

Supplement: S4 Fig — (TIF) [file pone.0235632.s004.tif]

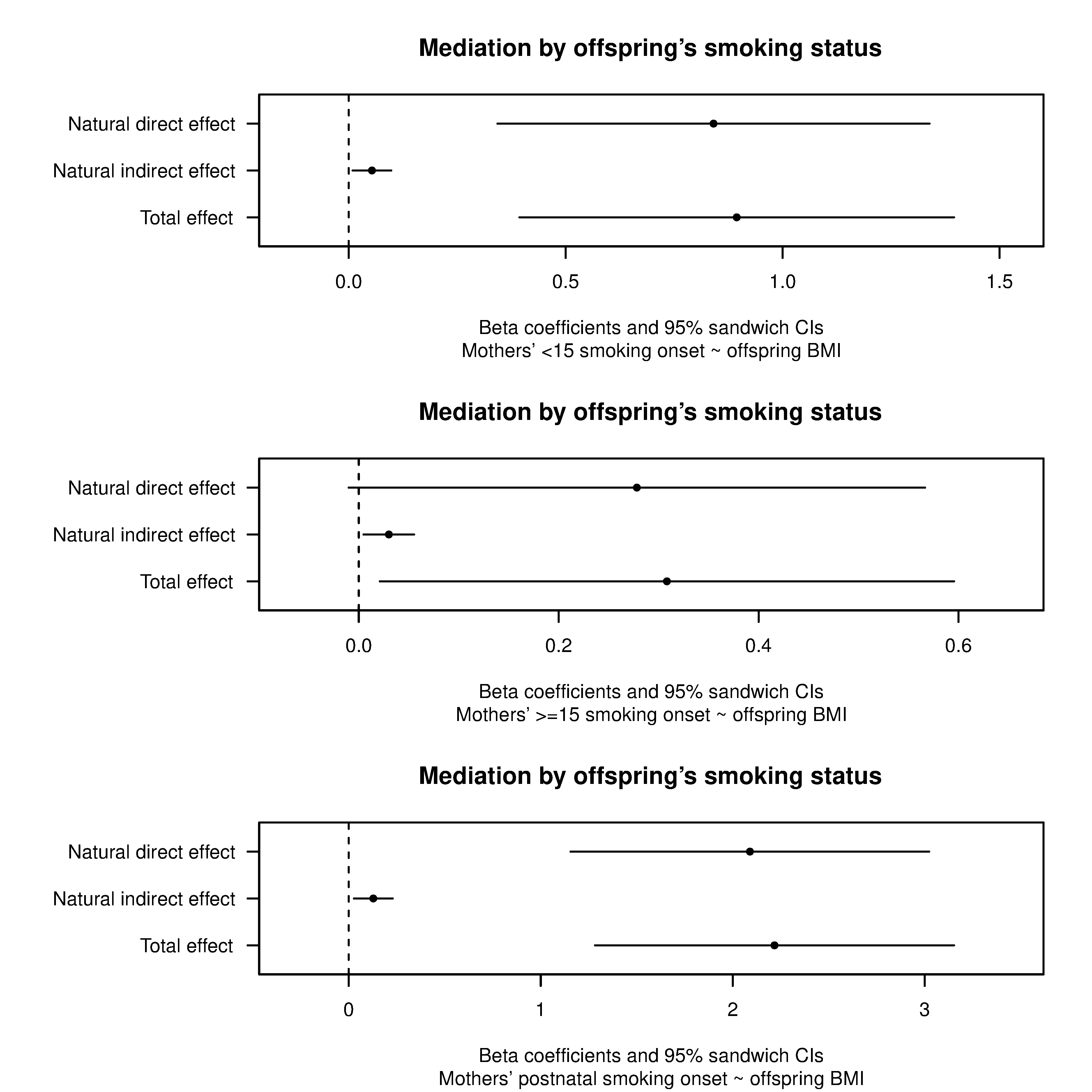

Supplement: S5 Fig — (TIF) [file pone.0235632.s005.tif]

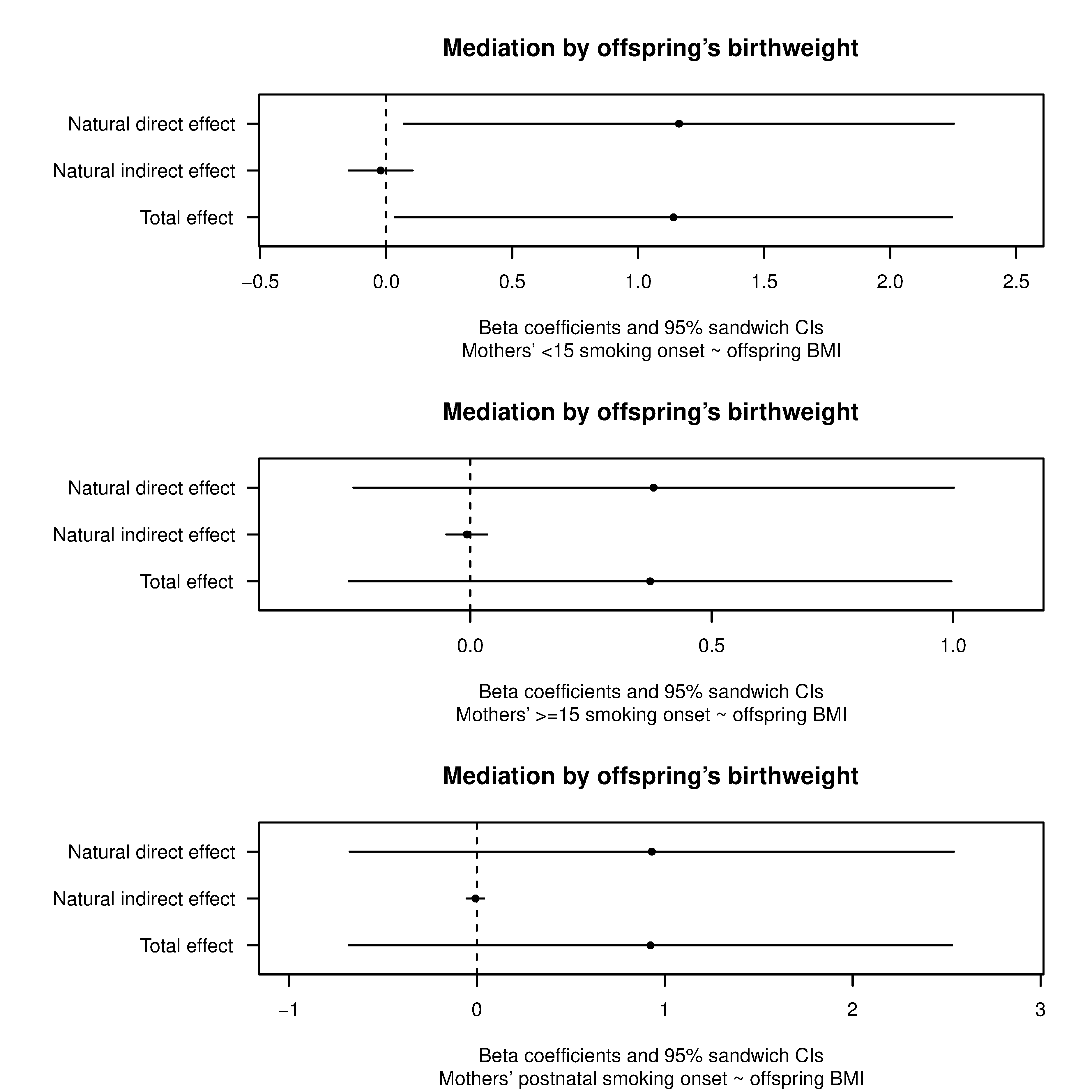

Supplement: S6 Fig — (TIF) [file pone.0235632.s006.tif]
