# Supplementary material for: Parents’ smoking onset before conception as related to body mass index and fat mass in adult offspring: Findings from the RHINESSA generation study
Source: PLoS One. 2020 Jul 6;15(7):e0235632. doi: 10.1371/journal.pone.0235632 (PMC7337347; doi:10.1371/journal.pone.0235632)
Supplement: S1 Table — A. Descriptive table of father offspring cohort grouped by fathers’ smoking onset and stratified by offspring sex. B. Descriptive table of mother offspring cohort grouped by mothers’ smoking onset and stratified by offspring sex. Parents who started smoking prior to conception have higher current BMI and less education compared to never smoking parents. Offspring of smoking parents have higher BMI, more frequently smoke themselves and have smoked more years, compared to offspring of never smoking parents. Sons with fathers who started smoking from age 15 but before conception also have higher FMI than sons with never smoking fathers. (PDF) [file pone.0235632.s007.pdf]

S2 Table A: Descriptive table of father offspring cohort grouped by fathers' smoking onset and stratified by offspring sex, N=2939

	**Never smoked**		**<15 smoking onset**		**≥ 15 smoking onset**		**Postnatal smoking onset**	
	Sons'	Daughters'	Sons'	Daughters'	Sons'	Daughters'	Sons'	Daughters'
**Father characteristics**	N=616 (49)	N=783 (47)	N=126 (10)	N=179 (11)	N=482 (38)	N=696 (41)	N=31 (3)	N=26 (2)
Age, mean ± SD	53.4 ± 6.5	53.9 ± 6.0	**57.4 ± 5.2**	**55.3 ± 6.1**	**56.7 ± 5.6**	**56.0 ± 5.7**	54.8 ± 5.8	56.0 ± 5.0
Range	39 - 65	40 - 65	42 – 65	41 – 65	40 - 65	39 - 66	45 - 64	47 - 64
Bmi kg/m^2, mean ± SD	26.5 ± 3.6	26.3 ± 3.7	**27.7 ± 3.8**	**27.7 ± 3.9**	**27.2 ± 4.1**	**27.1 ± 3.7**	26.6 ± 4.4	26.6 ± 2.3
Range	17 - 44	18 - 54	20 - 39	19 - 39	19 - 53	17 - 43	19 - 37	23 - 32
Primary education, n (%)	58 (9)	79 (10)	**38 (30)**	**58 (32)**	**89 (19)**	**127 (18)**	**1 (3)**	**3 (12)**
Secondary education	216 (35)	266 (34)	**45 (36)**	**78 (44)**	**188 (39)**	**263 (38)**	**17 (55)**	**10 (39)**
University/College	338 (55)	437 (56)	**42 (33)**	**42 (24)**	**195 (41)**	**300 (43)**	**13 (42)**	**13 (50)**
Years smoked, mean ± SD	-	-	**28.5 ± 13.6**	**27.0 ± 13.6**	**23.0 ± 13.5**	**22.5 ± 12.7**	**14.0 ± 9.9**	**14.2 ± 10.9**
Range			0 - 59	0 - 52	0 - 52	0 - 49	0 - 38	0 - 34
Packyears, median; 25,75th%								
From birth to offspring's age 18	-	-	**23.2; 14.5, 30**	**21.5; 11.7, 30**	**16.5; 8.2, 26.5**	**16.5; 10, 24**	**2.6; 0.8, 10**	**2.6; 2.0, 12**
In preconception years	-	-	**10.2; 7.3, 16.8**	**9.8; 5.2, 15**	**6; 3, 10**	**6.5; 3.8, 10**	-	-
Age smoke onset, mean ± SD	-	-	**13.4 ± 1.2**	**13.2 ±1.3**	**17.5 ± 2.6**	**17.3 ± 2.5**	**36.4 ± 8.9**	**34.9 ± 10.1**
Range			6 - 14	7 - 14	15 - 33	15 - 30	20 - 53	21 - 50
**Offspring characteristics**								
Age, mean ± SD	28 ± 7.1	28.4 ± 6.8	**31.3 ± 7.6**	**30.8 ± 7.5**	**30.9 ± 7.3**	**30.7 ± 7.2**	**32.6 ± 7.4**	**33.7 ± 7.9**
Range	18 - 49	18 - 49	18 - 47	18 - 50	18 - 48	18 - 50	20 - 47	19 - 48
Bmi kg/m^2, mean ± SD	24.6 ± 3.8	23.4 ± 4.5	25.3 ± 4.2	**24.6 ± 5.5**	**25.6 ± 4.4**	**24.1 ± 4.9**	25.6 ± 5.2	24.1 ± 4.8
Range	16 - 53	15 - 67	17 - 41	15 - 53	16 - 53	14 - 52	19 - 39	19 - 38
FMI fat mass kg/m^2, mean ± SD	3.0 ± 1.7	5.7 ± 2.4	4.1 ± 0.8	7.7 ± 3.7	**5.6 ± 3.4**	5.7 ± 2.1	5.7 ± 2.4	5.4 ± 1.4
Range	1.1 – 6.6	2.5 – 13.7	3.4 – 5.1	3.9 – 13.8	1.2 – 11.7	3.0 – 14.4	4.0 – 7.4	3.5 – 7.6
Primary education, n (%)	24 (4)	19 (2)	3 (2)	**3 (2)**	13 (3)	17 (2)	1 (3)	1 (4)
Secondary education	280 (46)	251 (32)	65 (52)	**79 (44)**	214 (50)	212 (31)	8 (26)	8 (31)
University/College	312 (51)	511 (65)	58 (46)	**97 (54)**	252 (52)	464 (67)	22 (71)	17 (65)
Never smoked, n (%)	476 (77)	606 (77)	**78 (62)**	**104 (58)**	**313 (65)**	**448 (64)**	**19 (61)**	**16 (62)**
Ever smoked	134 (22)	174 (22)	**48 (38)**	**72 (40)**	**169 (35)**	**247 (36)**	**12 (39)**	**10 (39)**
Years smoked, mean ± SD	7.6 ± 6.2	8.3 ± 6.5	**10.6 ± 7.2**	**10.4 ± 6.8**	**10.4 ± 7.2**	**9.3 ± 6.6**	9.8 ± 7.5	**14.0 ± 7.9**
Range	0 – 27	0 - 33	0 – 29	1 – 30	0 – 36	0 – 33	2 - 25	3 - 24
Age smoke onset, mean ± SD	17.1 ± 3.1	16.4 ± 2.8	**16.1 ± 3.1**	**15.4 ± 2.3**	16.8 ± 2.8	16.5 ± 2.8	17.8 ± 3.3	16.5 ± 2.4
Range	12 – 28	10 – 28	9 – 25	12 – 27	11 – 25	10 – 30	13 - 25	14 - 21

Missing values: Paternal characteristics: Age: NA=37; BMI: NA=34, Education level=23; Packyears: NA=836. Offspring characteristics: Age: NA=7; FMI: NA=2812; Education level: NA=8; Smoking-status: NA= 13; Years smoked: NA=72; Age smoking onset: NA=29.

Sign group differences <0.05 calculated by Wilcoxon and chi square/Kruskal test, marked in bold.

S2 Table B: Descriptive table of mother offspring cohort grouped by mothers' smoking onset and stratified by offspring sex, N=3548

	Never smoked		<15 smoking onset		≥ 15 smoking onset		Postnatal smoking onset	
	Sons'	Daughters'	Sons'	Daughters'	Sons'	Daughters'	Sons'	Daughters'
**Mother characteristics**	N=732 (48)	N=965 (47)	N=154 (10)	N=232 (12)	N=594 (39)	N=780 (39)	N=42 (3)	N=49 (3)
Age, mean ± SD	53.6 ± 6.7	53.8 ± 6.7	52.7 ± 6.4	**52.6 ± 6.1**	**55.4 ± 6.2**	**56.8 ± 6.0**	**57.6 ± 5.7**	**56.4 ± 6.2**
Range	39 - 65	39 - 65	40 - 65	40 - 65	39 - 65	39 - 65	42 – 65	40 - 64
Bmi kg/m^2, mean ± SD	25.1 ± 4.1	25.4 ± 4.2	**26.8 ± 5.1**	**27.1 ± 5.9**	25.4 ± 4.1	25.7 ± 4.6	**27.3 ± 4.7**	26.4 ± 4.8
Range	16 - 45	17 - 52	18 - 49	17 - 66	14 - 45	17 - 60	17 - 36	19 - 40
Primary education, n (%)	73 (10)	128 (13)	**29 (19)**	**62 (27)**	**85 (14)**	**161 (21)**	**10 (24)**	**10 (20)**
Secondary education, n (%)	238 (33)	288 (30)	**61 (40)**	**88 (38)**	**225 (38)**	**256 (33)**	**18 (43)**	**27 (55)**
University/College, n (%)	417 (57)	545 (57)	**62 (40)**	**81 (35)**	**280 (47)**	**361 (46)**	**14 (33)**	**12 (25)**
Years smoked, mean ± SD	-	-	**23.4 ± 13.6**	**24.6 ± 13.4**	**21.0 ± 13.3**	**20.6 ± 12.7**	**17.5 ± 11.5**	**17.6 ± 12.5**
Range			0 - 49	0 - 51	0 - 52	0 - 49	0 - 40	0 – 43
Packyears, median; 25,75th%								
From birth to offspring's age 18	-	-	**14; 8.5, 22.9**	**16.1; 10.2,23.9**	**12.6; 6, 18.8**	**12.5; 6, 8.8**	**4.5; 2.1, 6.7**	**3; 1.5, 7.9**
In preconception years	-	-	**5.5; 3, 9**	**6; 4, 9.6**	**4; 2.4, 6.6**	**4.5; 2.5, 7.5**	-	-
Age smoke onset, mean ± SD	-	-	**13.3 ± 0.9**	**13.4 ± 0.9**	**17.4 ± 2.3**	**17.3 ± 2.2**	**29.5 ± 8.1**	**29.2 ± 7.4**
Range			9 - 14	7 - 14	15 - 30	15 - 30	20 - 49	15 - 44
**Offspring characteristics**								
Age, mean ± SD	30.1 ± 7.7	30.3 ± 7.8	31.2 ± 8.5	**31.1 ± 8.0**	**31.5 ± 7.4**	31.0 ± 7.4	**39.1 ± 7.6**	**38.0 ± 7.4**
Range	18 - 50	18 - 51	19 - 50	19 - 52	18 - 49	18 - 50	19 - 52	23 - 51
Birthweight, mean ± SD	3.6 ± 0.5	3.4 ± 0.6	3.4 ± 0.6	**3.1 ± 0.7**	3.5 ± 0.7	3.4 ± 0.6	3.4 ± 0.6	3.5 ± 0.5
Range	1.1-5.0	0.5-5.3	2.0-4.4	1.0 – 4.3	1.1-5.3	0.5-4.8	2.3-4.4	2.5-4.3
Bmi kg/m^2, mean ± SD	24.7 ± 3.6	23.6 ± 4.4	**26.1 ± 4.6**	**24.6 ± 4.6**	**25.6 ± 3.9**	23.7 ± 4.2	**27.3 ± 3.3**	**26.1 ± 5.3**
Range	15 - 43	15 - 49	17 - 43	16 - 41	13 - 43	15 - 44	18 - 34	18 - 37
FMI fat mass kg/m^2, mean ± SD	4.9 ± 2.3	8.2 ± 4.8	3.6 -	7.5 ± 4.0	4.1 ± 2.3	6.7 ± 3.8	4.7 -	10.1 ± 5.2
Range	2.6 – 9.6	3.2 – 20.4	-	4.1 – 16.5	1.0 – 8.7	3.0 – 20.5	-	5.4 – 15.4
Primary education, n(%)	19 (3)	23 (2)	**6 (4)**	**10 (4)**	17 (3)	13 (2)	3 (7)	3 (2)
Secondary education, n(%)	307 (42)	304 (32)	**80 (52)**	**88 (38)**	243 (41)	240 (31)	20 (48)	19 (39)
university/College, n(%)	405 (55)	638 (66)	**68 (44)**	**133 (57)**	334 (56)	524 (67)	19 (45)	26 (53)
Never smoked, n (%)	511 (70)	700 (73)	**92 (60)**	**130 (56)**	398 (67)	**477 (61)**	**22 (52)**	**14 (29)**
Ever smoked, n (%)	218 (30)	262 (27)	**62 (40)**	**101 (44)**	193 (33)	**301 (39)**	**20 (48)**	**35 (71)**
Years smoked, mean ± SD	8.6 ± 7.0	8.5 ± 7.4	10.4 ± 7.4	**11.2 ± 7.2**	9.5 ± 6.4	**9.8 ± 6.7**	**15.6 ± 9.7**	**13.7 ± 8.6**
Range	0 – 32	0 – 35	0 – 37	1 – 30	0 – 28	0 – 32	2 - 36	0 - 33
Age smoke onset, mean ± SD	17.0 ± 3.0	16.6 ± 2.9	**15.6 ± 2.9**	**15.2 ± 3.1**	16.7 ± 3.3	**15.9 ± 2.3**	16.1 ± 3.5	16.1 ± 2.0
Range	7 – 27	10 – 28	10 – 24	10 – 36	8 – 32	11 – 25	8 – 25	12 - 20

Missing values: Maternal characteristics: Age: NA=80; BMI: NA=85; Education level: NA=17; Packyears: NA=868. Offspring characteristics: Age: NA=10; Birthweight: NA=2735; FMI: NA=3440; Education level: NA=6; Smoking-status: NA=12; Years smoked: NA=63; Age smoking onset: NA=25. Sign group differences, <0.05 calculated by Wilcoxon and chi square/Kruskal test, marked in bold
