# Supplementary material for: Parents’ smoking onset before conception as related to body mass index and fat mass in adult offspring: Findings from the RHINESSA generation study
Source: PLoS One. 2020 Jul 6;15(7):e0235632. doi: 10.1371/journal.pone.0235632 (PMC7337347; doi:10.1371/journal.pone.0235632)
Supplement: S2 Table — The figure shows regression model adjusted for mothers’ education and offspring sex and reveals no association with mothers’ preconception and postnatal smoking onset and FMI in her offspring. (PDF) [file pone.0235632.s008.pdf]

S3 Table: Associations between mothers' smoking onset and offspring (n=111) FMI

	Sons' and daughter's FMI		
Predictors	*Adj. difference in FMI (kg/m²)*	*95% CI*	*P*
Preconception smoking onset < 15 years of age	- 0.315	- 3.171 - 2.541	0.829
Preconception smoking onset ≥ 15 years of age	- 1.029	- 2.810 - 0.752	0.257
Postnatal smoking onset	1.947	- 2.207 - 6.102	0.358

Estimates from generalized linear regression models with adjustment for offspring sex and mothers' education. Clustered by family id and study centre. P value significance level: *.05, **.01, ***.001
