# Supplementary material for: Parents’ smoking onset before conception as related to body mass index and fat mass in adult offspring: Findings from the RHINESSA generation study
Source: PLoS One. 2020 Jul 6;15(7):e0235632. doi: 10.1371/journal.pone.0235632 (PMC7337347; doi:10.1371/journal.pone.0235632)
Supplement: S3 Table — The association between mothers’ preconception smoking onset and offspring BMI is fully mediated by mothers’ postnatal pack years, whereas mothers’ postnatal smoking onset and offspring BMI is partially mediated by mothers’ postnatal packyears. There is no evidence of direct or indirect effects via mothers’ preconception accumulative smoking in relation to mothers’ smoking onset. (PDF) [file pone.0235632.s009.pdf]

S4 Table: Mothers' pack years as mediator of the observed associations between mothers' smoking onset and offspring BMI

Causal mediation analysis mother offspring				
Mothers' smoking onset	*Adj diff. BMI (kg/m²)*	*Std. error*	*z value*	*P value*
**A) Mediation by mothers' packyears up to offspring age 18**				
Preconception smoking onset <15				
Natural direct effect	0.228	0.421	0.540	0.589
Natural indirect effect	1.059	0.253	4.193	< 0.000 ***
Total effect	1.287	0.349	3.692	< 0.000 ***
Interaction by offspring sex: 0.774				
Preconception smoking onset ≥15				
Natural direct effect	- 0.299	0.256	- 1.165	0.244
Natural indirect effect	0.833	0.199	4.178	< 0.000 ***
Total effect	0.534	0.193	2.776	0.006 **
Interaction by offspring sex : 0.542				
Postnatal smoking onset				
Natural direct effect	1.950	0.541	3.608	< 0.000 ***
Natural indirect effect	0.276	0.080	3.462	< 0.000 ***
Total effect	2.226	0.540	4.120	< 0.000 ***
Interaction by offspring sex: 0.743				
**B) Mediation by mothers' preconception packyears**				
Preconception smoking onset <15				
Natural direct effect	0.580	0.342	1.694	0.090
Natural indirect effect	0.291	0.181	1.607	0.108
Total effect	0.870	0.284	3.064	0.002 **
Interaction by offspring sex: 0.965				
Preconception smoking onset ≥15				
Natural direct effect	0.261	0.195	1.343	0.179
Natural indirect effect	0.191	0.120	1.594	0.111
Total effect	0.452	0.164	2.761	0.006 **
Interaction by offspring sex : 0.966				

Effect decomposition on the scale of the linear predictor with standard errors based on the sandwich estimator. Conditional on fathers' educational level and offspring sex.
P value significance level: * 05, ** 01, *** 001
