# Supplementary material for: Parents’ smoking onset before conception as related to body mass index and fat mass in adult offspring: Findings from the RHINESSA generation study
Source: PLoS One. 2020 Jul 6;15(7):e0235632. doi: 10.1371/journal.pone.0235632 (PMC7337347; doi:10.1371/journal.pone.0235632)
Supplement: S4 Table — The association between mothers’ preconception smoking onset before 15 years of age as well as smoking onset after birth and offspring BMI is partially mediated by mothers’ BMI. There is no evidence of direct or indirect effects via mothers’ BMI in relation to mothers’ preconception smoking onset ≥15. (PDF) [file pone.0235632.s010.pdf]

S5 Table: Mothers' BMI as mediator of the observed associations between mothers' smoking onset and offspring BMI

Causal mediation analysis mother offspring				
Mothers' smoking onset	*Adj diff. BMI (kg/m²)*	*Std. error*	*z value*	*P value*
**Mediation by mothers' BMI**				
Preconception smoking onset <15				
Natural direct effect	0.551	0.247	2.229	0.026 *
Natural indirect effect	0.334	0.074	4.483	< 0.000 ***
Total effect	0.884	0.258	3.436	< 0.000 ***
Interaction by offspring sex: 0.827				
Preconception smoking onset ≥15				
Natural direct effect	0.258	0.143	1.809	0.070
Natural indirect effect	0.023	0.039	0.596	0.551
Total effect	0.282	0.149	1.896	0.058
Interaction by offspring sex : 0.912				
Postnatal smoking onset				
Natural direct effect	1.869	0.495	3.774	< 0.000 ***
Natural indirect effect	0.320	0.128	2.496	0.013 *
Total effect	2.188	0.486	4.500	< 0.000 ***
Interaction by offspring sex: 0.354				

Effect decomposition on the scale of the linear predictor with standard errors based on the sandwich estimator. Conditional on fathers' educational level and offspring sex.
P value significance level: *.05, **.01, ***.001
