# Supplementary material for: Parents’ smoking onset before conception as related to body mass index and fat mass in adult offspring: Findings from the RHINESSA generation study
Source: PLoS One. 2020 Jul 6;15(7):e0235632. doi: 10.1371/journal.pone.0235632 (PMC7337347; doi:10.1371/journal.pone.0235632)
Supplement: S5 Table — The association between mothers’ preconception smoking onset before 15 years of age as well as smoking onset after birth and offspring BMI is partially mediated by offspring’s own smoking status. (PDF) [file pone.0235632.s011.pdf]

S6 Table: Offspring's smoking habits as mediator of the observed associations between mothers' smoking onset and offspring BMI

Causal mediation analysis mother offspring				
Mothers' smoking onset	*Adj diff. BMI (kg/m²)*	*Std. error*	*z value*	*P value*
**Mediation by sons' and daughters' ever/never smoking**				
Preconception smoking onset <15				
Natural direct effect	0.841	0.254	3.310	< 0.000 ***
Natural indirect effect	0.059	0.024	2.411	0.016 *
Total effect	0.899	0.256	3.518	< 0.000 ***
Interaction by offspring sex: 0.767				
Preconception smoking onset ≥15				
Natural direct effect	0.278	0.147	1.892	0.059
Natural indirect effect	0.031	0.013	2.352	0.019 *
Total effect	0.309	0.147	2.108	0.035 *
Interaction by offspring sex : 0.335				
Postnatal smoking onset				
Natural direct effect	2.090	0.476	4.386	< 0.000 ***
Natural indirect effect	0.129	0.052	2.491	0.013 *
Total effect	2.219	0.477	4.652	< 0.000 ***
Interaction by offspring sex: 0.512				

Effect decomposition on the scale of the linear predictor with standard errors based on the sandwich estimator. Conditional on fathers' educational level and offspring sex.
P value significance level: *.05, **.01, ***.001
