# Supplementary material for: Parents’ smoking onset before conception as related to body mass index and fat mass in adult offspring: Findings from the RHINESSA generation study
Source: PLoS One. 2020 Jul 6;15(7):e0235632. doi: 10.1371/journal.pone.0235632 (PMC7337347; doi:10.1371/journal.pone.0235632)
Supplement: S6 Table — In a subsample with birth weight data, there is no evidence of mediation by offspring birthweight. (PDF) [file pone.0235632.s012.pdf]

S7 Table: Offspring's birthweight as mediator of the observed associations between mothers' smoking onset and offspring BMI

Causal mediation analysis mother offspring				
Mothers' smoking onset	*Adj diff. BMI (kg/m²)*	*Std. error*	*z value*	*P value*
**Mediation by sons' and daughters' birthweight**				
Preconception smoking onset <15				
Natural direct effect	1.162	0.557	2.087	0.037 *
Natural indirect effect	- 0.021	0.062	- 0.338	0.736
Total effect	1.141	0.564	2.025	0.043 *
Interaction by offspring sex: 0.329				
Preconception smoking onset ≥15				
Natural direct effect	0.380	0.317	1.196	0.232
Natural indirect effect	- 0.008	0.025	- 0.328	0.743
Total effect	0.372	0.319	1.165	0.244
Interaction by offspring sex : 0.273				
Postnatal smoking onset				
Natural direct effect	0.932	0.820	1.136	0.256
Natural indirect effect	- 0.008	0.025	- 0.315	0.753
Total effect	0.924	0.819	1.128	0.259
Interaction by offspring sex: 0.362				

Effect decomposition on the scale of the linear predictor with standard errors based on the sandwich estimator. Conditional on fathers' educational level and offspring sex. Sub-sample with n = 813 offspring
P value significance level: *.05, **.01, ***.001
