# Supplementary material for: Parents’ smoking onset before conception as related to body mass index and fat mass in adult offspring: Findings from the RHINESSA generation study
Source: PLoS One. 2020 Jul 6;15(7):e0235632. doi: 10.1371/journal.pone.0235632 (PMC7337347; doi:10.1371/journal.pone.0235632)
Supplement: S1 File — (PDF) [file pone.0235632.s013.pdf]

S1 Resource. Ethic committee name and approval number for each study center:

Australia, Melbourne: Monash University Human Research Ethics Committee Project # CF11/1818-2010001012

Belgium, South Antwerp and Antwerp City: Comité voor Medische Ethiek UZA/UA 11/41/288 – UA

Denmark, Aarhus: De Videnskabsetiske Komiteer for region Midtjylland. M-20110106

Estonia, Tartu: Research Ethics Committee of the University of Tartu (UT REC) 209T-17 and 225/M-24

France, Paris: Etude ECRHS III: promotion CHU de Grenoble. Ethical approval CPP Sud est V 4 mars 2011. Approval Ministry of Health AFSSAPS n°B110053-70.

France, Grenoble: Etude ECRHS III: promotion CHU de Grenoble. Ethical approval CPP Sud est V 4 mars 2011. Approval Ministry of Health AFSSAPS n°B110053-70.

France, Montpellier: Etude ECRHS III : promotion CHU de Grenoble. Ethical approval CPP Sud est V 4 mars 2011. Approval Ministry of Health AFSSAPS n°B110053-70.

France, Bordeaux: Etude ECRHS III : promotion CHU de Grenoble. Ethical approval CPP Sud est V 4 mars 2011. Approval Ministry of Health AFSSAPS n°B110053-70.

Germany, Hamburg: Ethikkommission der Bayerischen Landesärztekammer (Positive Votum: 10015

Germany, Erfurt: Ethikkommission der Bayerischen Landesärztekammer (Positive Votum: 10015

Iceland, Reykjavik: National Bioethics committee of Iceland VSN-11-121-S3

Norway, Bergen: Regional Ethics Committee West Norway 2010/759

Spain, Barcelona: Ethics Committee of the Parc de Salut Mar, Barcelona (Comité del etico d'investigacion clínica (CEIC)- Parc de Salut Mar, Barcelona (Approval number) 2009/3500/1

Spain, Galdakao: Ethics Committee of the Parc de Salut Mar, Barcelona (Comité etic d'investigacio clínica (CEIC)- Parc de Salut Mar, Barcelona (Approval num) 2009/3500/1

Spain, Albacete: Ethics Committee of the Parc de Salut Mar, Barcelona (Comité etic d'investigacio clínica (CEIC)- Parc de Salut Mar, Barcelona (Approval number) 2009/3500/1

Spain, Oviedo: Ethics Committee of the Parc de Salut Mar, Barcelona (Comité del etico d'investigacion clínica (CEIC)- Parc de Salut Mar, Barcelona (Approval number) 2009/3500/1

Spain, Huelva: Ethics Committee of the Parc de Salut Mar, Barcelona (Comité del etico d'investigacion clínica (CEIC)- Parc de Salut Mar, Barcelona (Approval number) 2009/3500/1

Sweden, Gothenburg: Regional Ethical Review Board in Uppsala. 2010/432

Sweden, Uppsala: Regional Ethical Review Board in Uppsala. And the number of the decision is 2010/432

Sweden, Umeaa: Regional Ethical Review Board in Uppsala. 2010/432

UK, Ipswich: NRES committee London-Stanmore REC Ref: 11/LO/0965

UK, Norwich: NRES committee London-Stanmore REC Ref: 11/LO/0965
